# Acute Effects of Stimulant Medication on Gray Matter and White Matter Indices in Healthy Controls and Adults With ADHD

**DOI:** 10.1002/brb3.71606

**Published:** 2026-07-16

**Authors:** Thunberg Per, Msghina Mussie

**Affiliations:** ^1^ Faculty of Medicine and Health Centre for Experimental and Biomedical Imaging in Örebro (CEBIO) Örebro University Örebro Sweden; ^2^ Department of Radiology and Medical Physics Faculty of Medicine and Health Örebro University Örebro Sweden; ^3^ Department of Psychiatry School of Medical Sciences Faculty of Medicine and Health Örebro University Örebro Sweden; ^4^ Department of Clinical Neuroscience Karolinska Institutet Stockholm Sweden

**Keywords:** ADHD, diffusion‐tensor imaging, stimulant medication, voxel‐based morphometry

## Abstract

**Introduction:**

Short‐latency changes in gray matter volume (GMV) and diffusion tensor imaging (DTI) indices have been reported for sleep deprivation, hypercapnia, short‐lasting visual and nociceptive stimuli and within a couple of hours after ingestion of single‐dose psychoactive drugs.

**Methods:**

In the current study, we investigated acute effects of single‐dose stimulant medication (CS) on gray and white matter indices using voxel‐ and surface‐based morphometry (VBM and SBM) and diffusion tensor imaging (DTI) in 78 participants, consisting of 42 healthy controls and 36 age and gender matched adults with ADHD. Heart rate and blood pressure were also measured under similar conditions as physiological covariates.

**Results:**

For the group as a whole (*n* = 78), acute ingestion of CS caused apparent increase in GMV in bilateral operculum, insula, cingulate and paracingulate cortices, fusiform gyrus, superior, middle and inferior gyri of the temporal cortex and in right putamen and caudate nucleus and caused an even more wide‐spread right‐lateralized apparent increase in cortical thickness; the apparent changes in cortical thickness were also seen in patients with ADHD but not in healthy controls. CS administration also increased fractional anisotropy (FA) in bilateral corpus collosum, corona radiata and right superior longitudinal fasciculus, with no significant differences in VBM, SBM or FA between healthy controls and ADHD patients before or after CS ingestion. Heart rate and blood pressure increased significantly for both groups after CS ingestion, with ADHD patients displaying greater increase in systolic blood pressure compared to healthy controls. No significant correlation was detected between the structural gray matter and white matter changes and changes in heart rate or blood pressure.

**Conclusion:**

Combining VBM, SBM and DTI indices, our study is the first to show apparent structural changes in the brain in adults with ADHD within 1–2 h after CS ingestion. Currently it is not known what causes these apparent structural changes but notwithstanding that, these changes should be taken into account in longitudinal VBM, SBM and DTI studies.

## Graphical abstract

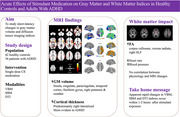

## 1 Introduction

Attention deficit hyperactivity disorder (ADHD) is a neurodevelopmental disorder that is believed to affect roughly 6% of children and 3% of adults worldwide (Ginsberg et al. 2014) with core functional impairment characterized by difficulty in regulating attention and exerting top‐down inhibitory control over behavior. Recommended treatment is multi‐modal, with stimulant medication as the mainstay of pharmacological intervention (Cortese et al. 2018). Although by no means consistently found, numerous studies have reported structural and functional abnormalities in patients with ADHD compared to controls in children, adolescents and adults (Agoalikum et al. 2023). Although there are findings to the contrary, there are also studies suggesting that treatment with stimulant medication may potentially reverse some of the observed structural and functional abnormalities (Frodl and Skokauskas 2012).

More specifically, volume‐ and surface‐based morphometry (VBM and SBM) and diffusion‐tensor imaging (DTI) have been used to characterize gray matter and white matter abnormalities in ADHD. Results are somewhat inconsistent but systematic reviews and meta‐analysis of VBM studies have implicated several brain areas including fronto‐parietal, limbic, basal ganglia and cerebellar areas (Agoalikum et al. 2023), with some of these abnormalities more pronounced in non‐treated compared to CS treated patients (Frodl and Skokauskas 2012). Similar systematic reviews and meta‐analysis of cross sectional DTI studies have also revealed diffuse alterations in fronto‐striato‐cerebellar connections (Cui et al. 2023) and reduced fractional anisotropy (FA) in the body and splenium of the corpus callosum, implicating interhemispheric connections that serve cognitive and motor functions (Parlatini et al. 2023).

More recently, a growing number of studies have been reporting fast‐onset changes in gray and white matter measures after short‐lasting physiological stimuli and single‐dose pharmacological interventions. Acute effects of the D2 receptor antagonist haloperidol (Tost et al. 2010) and the GABA b receptor agonist baclofen (Franklin et al. 2013) as well as short‐lasting visual (Månsson et al. 2020; Naegel et al. 2017) and nociceptive stimuli (Broessner et al. 2021) have been reported to produce immediate changes in gray matter indices. Using multimodal pharmaco‐neuroimaging, Tost et al. (2010) found that D2 receptor blockade with single‐dose haloperidol induced reversible striatal volume changes within hours of drug ingestion, which were correlated with acute extrapyramidal motor symptoms. Franklin et al. (2013) found that a single dose of the GABA b agonist baclofen led to apparent reductions in gray matter volume in the dorsal and rostral anterior cingulate cortex. Månsson et al. (2020) reported that visual stimulation with alternating checkerboard led to instant, short‐lasting alterations of VBM indices in primary and secondary visual areas. Similar findings were reported by Naegel et al. (2017) who found changes in gray matter volume and thickness in visual brain areas within 4 min after participants viewed pictures relative to a fixation cross. Broessner et al. (2021) found gray matter volume changes in central pain processing areas such as anterior cingulate and insular cortices following short‐lasting nociceptive stimuli, changes that were also seen after repetitive T1 imaging in the absence of nociception. Tsugawa et al. (2025) found that acute injection of moderate doses of alcohol enlarged ventricle volumes and reversibly reduce gray matter volumes. Breathing manipulations simulating hypercapnia and hyperoxia were also found to overestimate cortical thickness by 1.85% and gray matter volume by 3.32%, which was interpreted to be due to vasodilation and increased blood volume (Tardif et al. 2017). Regarding DTI, Elvsåshagen et al. (2019) found that a single night's sleep deprivation led to widespread fractional anisotropy (FA) decreases and reductions in axial diffusivity.

In the present paper, we extend such findings by studying the effects of single‐dose stimulant medication on gray and white matter indices using surface‐ and voxel‐based morphometry and DTI in 78 participants consisting of 42 healthy controls and 36 adults with ADHD. Our initial hypothesis, based on the Tost et al.’s (2010) findings, where the D2 receptor antagonist haloperidol reduced striatal volumes, was that stimulating D2 transmission with stimulant medication would increase VBM and SBM measures in dopamine innervated areas including the striatum; in other words, we expected stimulant medications to have the opposite effect of those seen with haloperidol in the paper by Tost et al. (2010).

## 2 Methods

### 2.1 Study Participants and Implementation

A total of 42 healthy controls and 36 ADHD patients were included in the study, which was approved by the Swedish Ethical Review Authority (Dnr 2020–02278; 2020–05590). Written consent was obtained from all participants in the study. The ADHD group was a well‐characterized clinical cohort that was recruited from the Neuropsychiatric Outpatient Clinic at Örebro University Hospital. Inclusion criteria for the ADHD cohort were (i) ADHD diagnosis obtained after extensive neuropsychiatric evaluation by a dedicated team of psychologists and senior consultants in psychiatry in accordance with Swedish guidelines, (ii) no ongoing psychosis, bipolar, depressive, substance use or sever autism spectrum disorder, (iii) no suicidal or aggressive behavior, and (iv) no contraindication for MRI investigation. The ADHD group consisted of 30 responders and 6 non‐responders to CS medication. Response and non‐response was determined by a score of 1 or 2 using clinician and patient‐rated Clinical Global Impression–Improvement (CGI‐I / PGI‐I, respectively). Choice of medication and treatment optimization was carried out by the treating physician and followed Swedish guidelines for the pharmacological treatment of ADHD (Läkemedelsverket 2016). The control group was recruited by advertising at a university campus and hospital area. Exclusion criteria for the healthy controls were (i) current or previous psychiatric and neurological ailment including substance use syndrome, (ii) ongoing psychoactive medication use, (iii) narrow‐angle glaucoma, (iv) oversensitivity to any of the two CS medications used in the study, and (v) incompatibility with magnetic resonance imaging (MRI). The CS medication used by the ADHD patients was either methylphenidate (MPH) or lisdexamfetamine (LDX) as selected and dose‐optimized by the treating physician.

All participants underwent MRI examinations before and after CS using the same MRI scanner and protocol settings. The first session was performed in the absence of CS and the second MRI session 1–2 h after ingestion of CS, which consisted of 30 mg short‐acting MPH for the healthy controls and MPH or LDX for the ADHD group after abstaining from CS medication for 24 h before the start of the first MRI session. CS was ingested directly after the end of the first session, and the second session started 1–2 h later to synchronize MRI examination with peak CS concentration in brain tissue.

### 2.2 Image Acquisition

All MRI examinations were performed on a 3 Tesla (3T) Signa Premier MR system using a 48 channel head/neck coil (GE HealthCare, WI, USA). A structural T1‐weighted image of the brain used for VBM and SBM analysis was acquired using a 3D, inversion recovery (IR)‐prepared, fast spoiled gradient‐recalled (SPGR) sequence (BRAVO): TR/TE/TI = 7.3/3/450 ms, slice thickness = 1.2 mm, acquired pixel sixe = 0.9×0.9 mm^2^, flip angle = 12°, and a reduction factor (ARC) of 2. The applied DTI pulse sequence consisted of a single shot spin‐echo sequence with echo‐planar imaging using the following parameters: TR = 8000 ms, TE∼56 ms (minimum), *b* = 1000 s/mm^2^ and 15 encoding directions, 3 reference images (*b* = 0 s/mm^2^), field‐of‐view (FOV) = 26 cm, acquisition matrix = 128×128, 60 contiguous 2.5 mm slices (no slice gap), and an acceleration factor (ASSET) of 2. Real‐time field adjustment was enabled in order to reduce image distortion and misregistration and the “Super G” option was chosen to employ maximum gradient amplitudes.

### 2.3 VBM and SBM Processing

The CAT12 software (version 12.9) was used for VBM and SBM processing of the structural images (Gaser et al. 2024). The longitudinal processing pipeline was employed for VBM for detection of small brain changes (e.g., brain plasticity) and from SBM the cortical thickness was measured. Pre‐processed images were smoothed using a kernel with 6 mm and 15 mm FWHM for VBM and SBM thickness data, respectively.

### 2.4 DTI Processing

All images were eddy current and motion corrected using ECMOCO software (Mohammadi et al. 2010). Fractional anisotropy (FA) and mean diffusivity (MD) images were calculated using dtifit as part of FSL (ver. 6.0.4) (Smith et al. 2004). Voxel wise statistical analysis of the FA and MD data was carried out using TBSS (Tract‐Based Spatial Statistics, Smith et al. 2006; also part of FSL, Smith et al. 2004). TBSS projects all subjects' FA and MD data onto a mean FA tract skeleton, before applying voxel wise cross‐subject statistics.

For each subject, the mean and pre‐ versus post‐CS difference of FA and MD images, respectively, were calculated and later used in the statistical analysis.

### 2.5 Statistical Analysis

All statistical analysis of non‐image data was performed using R (release 4.5.1). A 2 × 2 repeated‐measures permutation ANOVA was conducted to assess the effect of group (healthy controls vs. ADHD patients), time (pre‐ vs. post‐CS ingestion) and interaction (group × time) having heart rate and systolic and diastolic blood pressure as dependent variables, respectively. Each analysis included 5000 permutations using aovperm from the permuco package. Group values of heart rate and blood pressure are reported using the median and interquartile range (IQR), while mean value and one standard deviation is used for age. The Wilcoxon rank‐sum test was used for statistical inference when comparing two groups with each other.

The analysis of VBM and SBM data was performed using statistical models available in the CAT12 software, while FA and MD image analysis was carried out using FSL:s randomize function (v2.9). A 2 × 2 repeated‐measures ANOVA model was applied to the VBM, SBM, FA and MD data sets, respectively, to study main effects of group (healthy controls and ADHD patients) and time (pre‐ and post‐CS) as well as interaction (group × time). We consistently used Threshold‐Free Cluster Enhancement (TFCE) with 5000 permutations in all image analysis to correct for multiple comparisons using false discovery rate (FDR) and a statistical significance set at *p* < 0.05.

## 3 Results

Mean age of all included participants (*n* = 78) was 35 ± 10 and consisted of 47 females and 31 males. There were no significant differences between the healthy controls (*n* = 42) and ADHD patients (*n* = 36) regarding age (34 ± 11 and 36 ± 9 years, respectively) or gender distribution (26/16 and 21/15, female/male, respectively). There were 9 missing values for heart rate and blood pressure scores, 3 in the control group and 6 in patients with ADHD.

### 3.1 Effect of Stimulant Medication on Heart Rate and Blood Pressure

Heart rate and blood pressure for controls and patients with ADHD are shown in Table 1. Repeated‐measures ANOVA revealed significant main group effect regarding heart rate (*p* = 0.004), with ADHD patients having a significant higher heart rate at baseline compared to controls. There was significant main effect of medication, which increased heart rate (*p* = 0.0002) as well as systolic (*p* = 0.001) and diastolic (*p* = 0.02) blood pressure. An interaction effect for systolic blood pressure (*p* = 0.03) was found with ADHD patients displaying significantly larger increment in this compared to healthy controls.

**TABLE 1:** Heart rate and blood pressure for healthy controls and ADHD patients before and after CS ingestion.

		All *n* = 69	Control *n* = 39	ADHD *n* = 30	*p*‐value*
Pre	Systolic blood pressure (mmHg)	125 (16)	124 (15)	126 (16)	NS
	Diastolic blood pressure (mmHg)	82 (11)	81 (12)	83.5 (9.5)	NS
	Heart rate (bpm)	70 (12)	64 (11.5)	74.5 (10)	<0.001
Post	Systolic blood pressure (mmHg)	129 (19)	129 (16)	136.5 (15.5)	NS
	Diastolic blood pressure (mmHg)	87 (12)	86 (12)	88.5 (11.25)	NS
	Heart rate (bpm)	82 (22)	78 (24)	87.5 (18.75)	0.03

*Wilcoxon rank‐sum test of control vs. ADHD.

*Note*: Values are presented as median (IQR).

NS = not significant.

### 3.2 Effect of Stimulant Medication on VBM and SBM Indices

**FIGURE 1:**
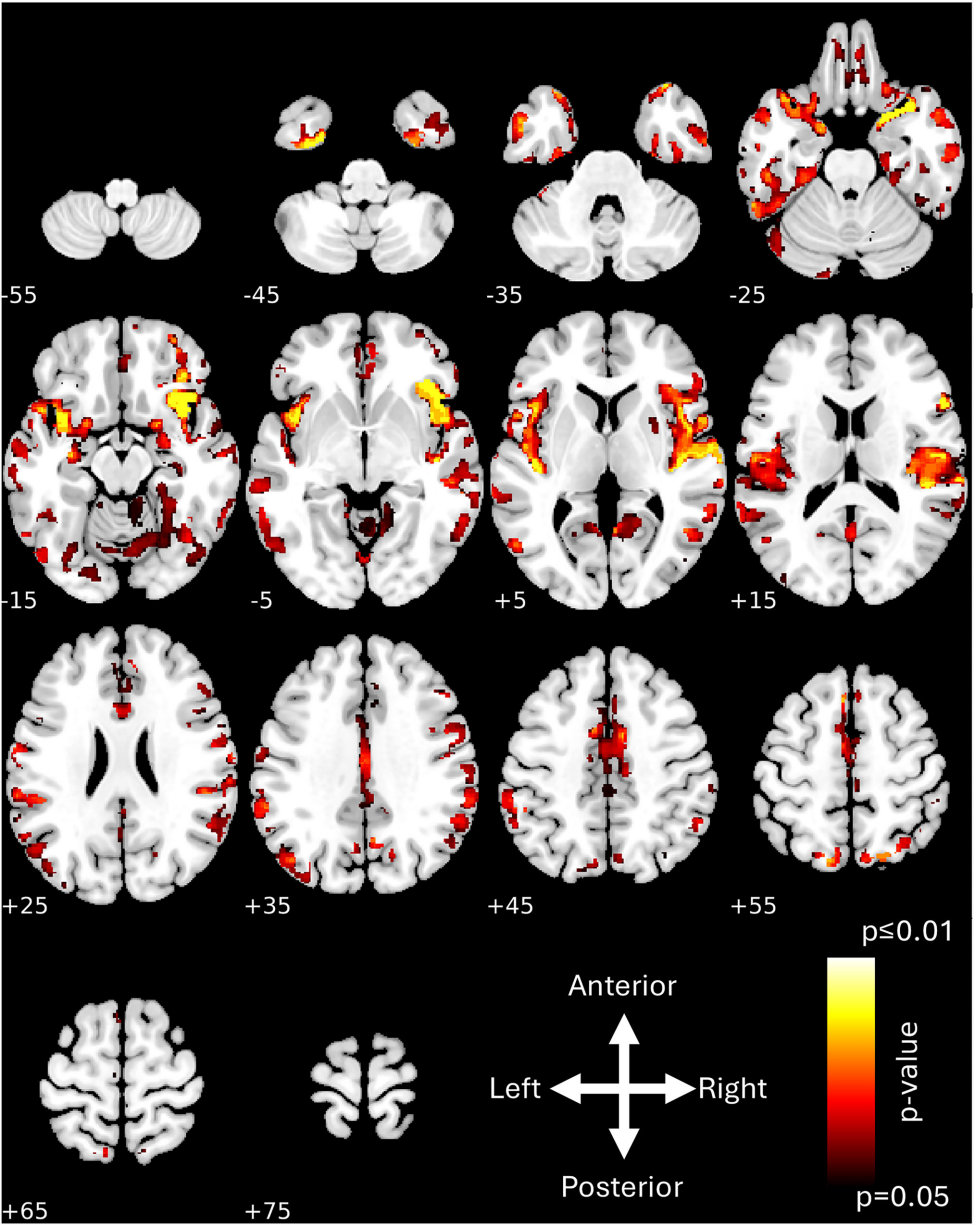
Axial slices showing areas with significant increment in VBM indices, as a main effect of CS administration for both healthy controls and ADHD patients taken as a whole (*n* = 78).

**FIGURE 2:**
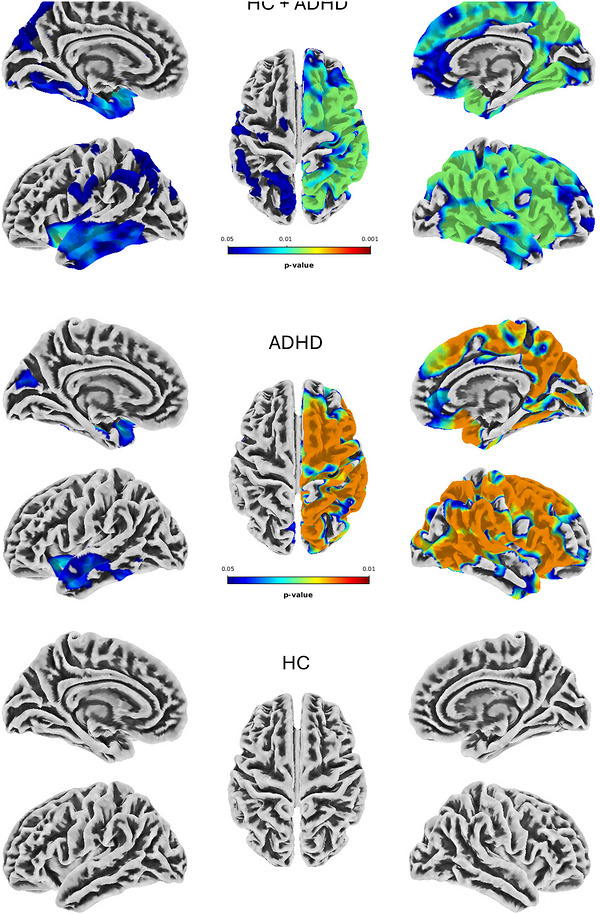
Cortical regions with significant increased cortical thickness after administration of CS shown on an inflated brain surface for the group as a whole (*n* = 78, upper panel), ADHD patients (*n* = 36, middle panel), and healthy controls (*n* = 42, lower panel).

Increased gray matter indices in both VBM and SBM were seen 1–2 h after CS‐administration as main effect for the group as a whole (*n* = 78) and in SBM as treatment effect for patients with ADHD (*n* = 36), but none in healthy controls (*n* = 42). For VBM, large bilateral changes were seen in regions encompassing opercular (central, frontal, temporal, and parietal) and insular cortex, cingulate (anterior and posterior), paracingulate, fusiform (temporal and occipital lobe) and superior, middle, and inferior temporal gyri. In the basal ganglia, there was increased VBM indices after CS administration in right putamen and caudate nucleus (Figure 1). As shown in Figure 2, significant right‐lateralized increase in SBM measures were found in widely distributed brain areas for the group as a whole (upper panel) and in patients with ADHD (middle panel), but not healthy controls (lower panel). Regions with SBM increases after CS administration are shown in the supplement using the Desikan‐Killiany (DK40) and Destrieux atlas (2009) surface‐based brain atlases (Table S1 and Figure S1). There was no significant main effect of group or interaction of group by treatment for any of the structural gray matter measures.

### 3.3 Effect of Stimulant Medication on DTI Measures

**FIGURE 3:**
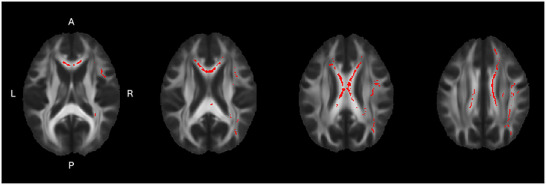
Axial slices showing the mean fractional anisotropy (FA) calculated from all examinations. Red regions show significant main effect of CS treatment resulting in an increased FA.

We found a main effect for the group as a whole of CS administration with apparent increase in FA in several brain regions including the corpus collosum (symmetric), anterior corona radiata (symmetric) and right superior longitudinal fasciculus (Figure 3). There were no significant main effect or interaction in mean diffusivity (MD).

## 4 Discussion

Structural T1‐weighted images for gray matter volume (VBM) and surface (SBM) measures and diffusion tensor imaging (DTI) are widely used to characterize structural integrity in gray and white matter brain areas, and differences in these indices are generally assumed to correctly reflect frank differences in gray matter volume/thickness and white matter integrity, respectively (Beaulieu 2002). Using these methods, in the present paper we report (i) more or less symmetrical volumetric changes, (ii) largely right‐lateralized widespread cortical thickness changes, and (iii) symmetrical increases in fractional anisotropy (FA) in the corpus callosum and anterior corona radiata 1–2 h after ingestion of single‐dose stimulant medication. Change in all three structural indices were noted for the sampled population as a whole (*n* = 78), which consisted of 42 healthy subjects and 36 adults with ADHD, and for SBM in patients with ADHD but not in healthy controls. This could either be due to lack of power or due to the know heterogeneity of the patient population, as we previously showed in another study (Thunberg et al. 2024). Physiological measures of peripheral nervous system activity—heart rate and systemic blood pressure—also increased 1–2 h after ingestion of stimulant medication, for the group as a whole and specifically for each sampled population, with significantly greater increase in heart rate and systolic blood pressure in patients with ADHD compared to healthy controls. Hemodynamic changes in cerebral blood pressure and blood flow have previously been hypothesized to lay behind the immediate short‐latency and transient longitudinal changes observed in VBM and SBM metrices (Ge et al. 2017; Naegel et al. 2017; Tardif et al. 2017). However, in the present study, we saw no correlation between the physiological (heart rate and blood pressure) and structural measures (VBM, SBM and FA), neither for the study group as a whole nor for the two sampled populations separately.

A number of studies have recently, rather surprisingly, reported fast‐onset changes in VBM, SBM and DTI measures after short‐lasting physiological stimuli or single‐dose pharmacological manipulation similar to that employed in the present paper. Currently, the basis for these short‐latency apparent structural changes in gray and white matter metrices is not fully understood, although different alternatives have been suggested. The observed changes do not seem to be experimental artifacts, as they occur in expected areas of the brain after hypothesis driven physiological stimulation and pharmacological intervention, for example, increased VBM and SBM measures in visual brain areas after short‐lasting visual stimuli (Månsson et al. 2020; Naegel et al. 2017), increases in VBM measures in central pain areas after short‐lasting nociceptive stimuli (Broessner et al. 2021), reductions in VBM measures in dopamine innervated areas such as the striatum 1–2 h after blockade of D2 receptors with the specific D2 antagonist haloperidol (Tost et al. 2010), reductions in VBM measures in densely GABA innervated areas of the prefrontal cortex such as the dorsal and rostral anterior cingulate cortex after stimulation of GABA b receptors with baclofen (Franklin et al. 2013), and wide‐spread reductions in FA after single‐night sleep deprivation that remained significant after adjusting for hydration measures (Elvsåshagen et al. 2019).

With some of these changes, however, occurring within as short as 4 min post‐stimulation, the preferred interpretation has shifted from frank structural remodeling of gray matter volume (Tost et al. 2010) and white matter integrity to putative cerebral fluid shifts and other similar hemodynamic factors (Naegel et al. 2017) and/or changes in brain metabolite concentrations (Ge et al. 2017). Our results are agnostic on this matter, other than showing lack of correlation between the changes in physiological and structural indices under our experimental conditions.

The pharmaco‐MRI paper by Tost et al. (2010) was of particular interest for us, as our initial hypotheses were based on it. We used stimulant medications that block reuptake of dopamine, which as a consequence of this enhance transmission through D2 receptors. We therefore expected our pharmacological intervention would have the opposite effect of blocking D2 receptors with haloperidol. As expected, we found increased VBM indices, among other things, in right putamen and caudate nucleus of the striatum, while Tost et al. (2010) reported reduced striatal volumes.

These apparent structural and diffusivity changes should be taken into account as putative group differences in GMV or FA could merely be a consequence of unaccounted for physiological stimuli or differential exposure to psychoactive drugs.

## 5 Limitations

Although the reported VBM, SBM and DTI changes for the group as a whole and changes in SBM for patients with ADHD were statistically quite robust and survived conservative corrections for multiple comparisons, we saw no significant difference between healthy controls and patients with ADHD when directly compared to each other, which potentially could be due to lack of power and limits the interpretation of our results. Second, lack of a placebo arm limits the specificity of the post‐medication structural changes, as these could also potentially be due to other non‐controlled experimental factors. Lastly, as was also the case in many of the other studies in the literature, the speed with which the changes in the structural indices occurred, that is, within 1–2 h after exposure to stimulant medication, makes interpretation in terms of genuine microstructural changes of gray and white matter rather untenable, unless the dynamics of microstructural remodeling of brain tissues is much faster than hitherto has been believed to be.

## 6 Conclusions

In the present paper, we show for the first time fast‐onset changes of gray and white matter indices as a result of single‐dose exposure of CS‐medication. Similar fast‐onset apparent changes in gray matter volume have previously been show for D2 antagonist and GABA b agonist. Future experiments including in animal models are needed to characterize the underlying nature of these fast‐onset changes.

## Author Contributions

**Thunberg Per**: conceptualization, investigation, writing – original draft, methodology, visualization, writing – review and editing, formal analysis, software. **Msghina Mussie**: conceptualization, investigation, funding acquisition, writing – original draft, methodology, validation, writing – review and editing, project administration, data curation, resources.

## Funding

This study is supported by Nyckelfonden, Örebro, Sweden (OLL 935421), ALF Grants, Region Örebro län, Sweden (OLL 973230).

## Conflicts of Interest

Authors have no competing interest to declare.

## Declaration of Generative AI and AI‐Assisted Technologies in the Writing Process

All materials presented in this work were created by the authors without any use of AI.

## Supporting information




**Supplementary Material**: brb371606‐sup‐0001‐SuppMat.docx

## Data Availability

Underlying deidentified data will be made available upon request to the corresponding author after approval of a proposal and signed agreement.
