# Supplementary material for: Acute Effects of Stimulant Medication on Gray Matter and White Matter Indices in Healthy Controls and Adults With ADHD
Source: Brain Behav. 2026 Jul 16;16(7):e71606. doi: 10.1002/brb3.71606 (PMC13376344; doi:10.1002/brb3.71606)
Supplement: Supplementary file 1 — Supplementary Material: brb371606‐sup‐0001‐SuppMat.docx [file BRB3-16-e71606-s001.docx]

**Supplement**

**Results from 2x2 repeated ANOVA using SBM data**

In the list below we report the regions, according to the Desikan-Killiany (DK40) and Destrieux (2009) brain atlases, where there was a significant (p<0.05 FDR corrected) main effect of time (pre and post CS) implying an increased cortical thickness. There were no significant main group or interaction effects.

Table S1 shows the regions with significant interaction effects using the Desikan-Killiany (DK40) and Destrieux (2009) atlases.

| **Brain atlas** | **Anatomic region** |
| --- | --- |
| *Desikan-Killiany (DK40)* | *Left hemisphere*  -insula  -superiortemporal  -middletemporal  -inferiortemporal  *Right hemisphere*  -transversetemporal  -bankssts  -supramarginal  -lingual  -insula  -lateralorbitofrontal  -superiortemporal  -superiorparietal  -parstriangularis  -inferiorparietal  - parsopercularis  -fusiform  -temporalpole  -postcentral  -precentral  -caudalmiddlefrontal  -isthmuscingulate  -precuneus  -superiorfrontal |
| *Destrieux atlas (2009)** | *Left hemisphere*  -G_temp_sup-Plan_polar  -S_circular_insula_inf  -S_circular_insula_ant  -G_Ins_lg_and_S_cent_ins  -G_temporal_middle  -G_subcallosal  -S_orbital_med-olfact  -G_parietal_sup-G_temporal_inf  *Right hemisphere*  - G_pariet_inf-Supramar  -S_circular_insula_ant  -G_temp_sup-Plan_polar  -G_and_S_subcentral  -S_interm_prim-Jensen  -G_pariet_inf-Angular  -G_temp_sup-G_T_transv  -S_orbital_med-olfact  -S_collat_transv_post  -G_insular_short  -S_oc-temp_lat  -G_front_inf-Opercular  -S_intrapariet_and_P_trans  -S_circular_insula_sup  -G_front_inf-Triangul  -G_precuneus  -G_subcallosal  -G_cingul-Post-ventral  -G_oc-temp_lat-fusifor  -G_oc-temp_med-Lingual  -S_circular_insula_inf  -G_parietal_sup  -S_front_inf  -G_orbital |

* G_ stands for Gyrus and S_ for Sulcus


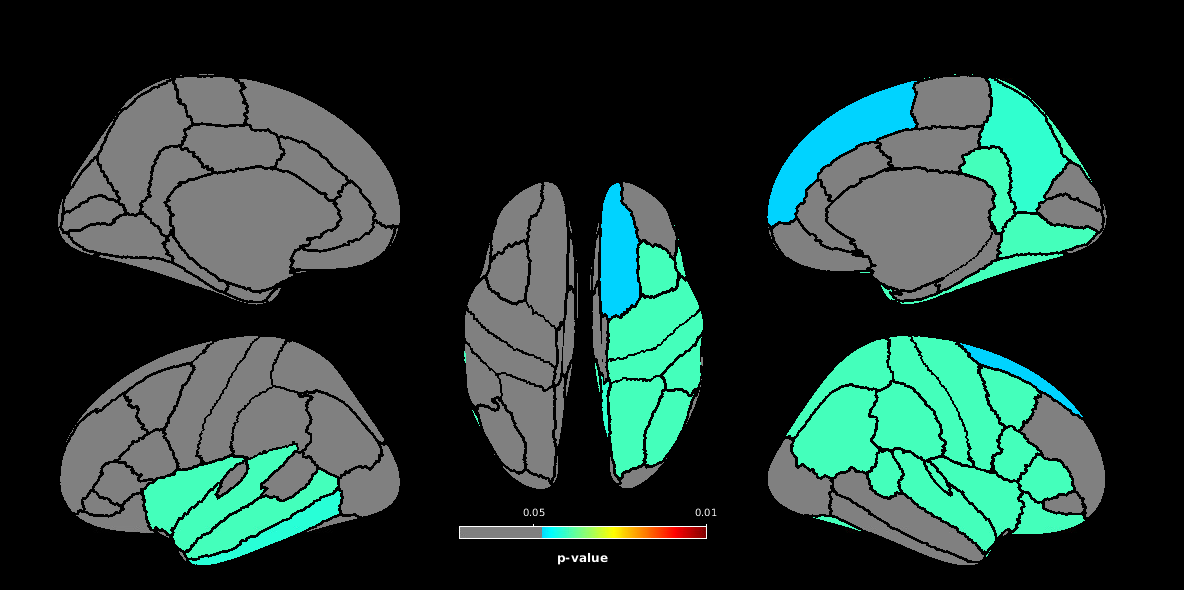


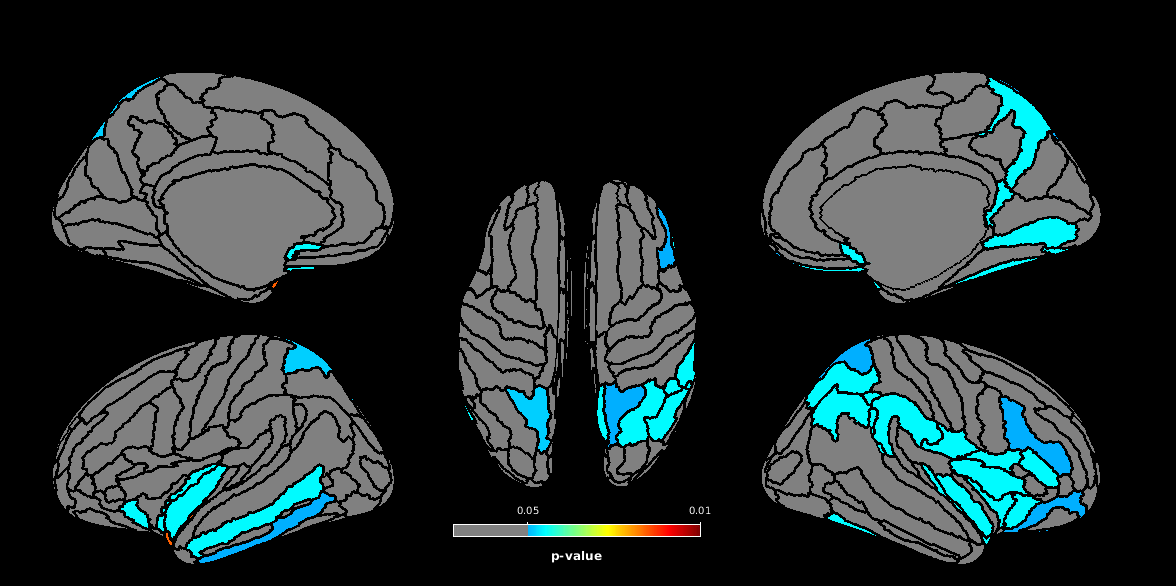


**Figure S1**. Significant regions with increased cortical thicknesses after CS according to the Desikan-Killiany (DK40) (above) and Destrieux 2009 (below) atlases.
